# Amlodipine-Induced Gingival Hypertrophy: A Case Report

**DOI:** 10.7759/cureus.35540

**Published:** 2023-02-27

**Authors:** Karthik Rajaram Mohan, Saramma Mathew Fenn, Ravikumar Pethagounder Thangavelu, Mohithan Subramaniam

**Affiliations:** 1 Oral Medicine and Radiology, Vinayaka Mission's Sankarachariyar Dental College, Vinayaka Mission's Research Foundation (Deemed to be University), Salem, IND

**Keywords:** gingivectomy, antihypertensive drugs, oral health care, amlodipine, gingival hypertrophy

## Abstract

Gingival hypertrophy caused by certain drugs, including amlodipine, may occur in genetically susceptible individuals. There is no clear explanation for the exact mechanism behind gingival hypertrophy, but a multifactorial theory has been proposed that unifies the phenomenon. In addition to causing difficulty with speech and mastication, gingival hypertrophy also contributes to poor oral hygiene and unaesthetic appearance. We describe the case of a 54-year-old woman who developed gingival hypertrophy due to the long-standing antihypertensive medication amlodipine 5 mg taken twice daily for four years.

## Introduction

The term "gingival overgrowth" refers to the overgrowth of the gingival tissues around the teeth. Gingival overgrowth is a common adverse effect of some drugs not prescribed for dental use, including anticonvulsants (particularly phenytoin), immunosuppressants (mainly cyclosporine), and calcium channel blockers (mainly nifedipine). Other synonyms for gingival overgrowth include gingival hyperplasia, gingival hypertrophy, and hyperplastic gingivitis. In similar ways, these drugs influence the periodontium, particularly the gingival connective tissue, leading to gingival overgrowth. Our case report discusses a 54-year-old female with hypertension who developed amlodipine-induced gingival overgrowth as a result of 5 mg amlodipine drug use twice daily for four years. 

## Case presentation

**Figure 1:**
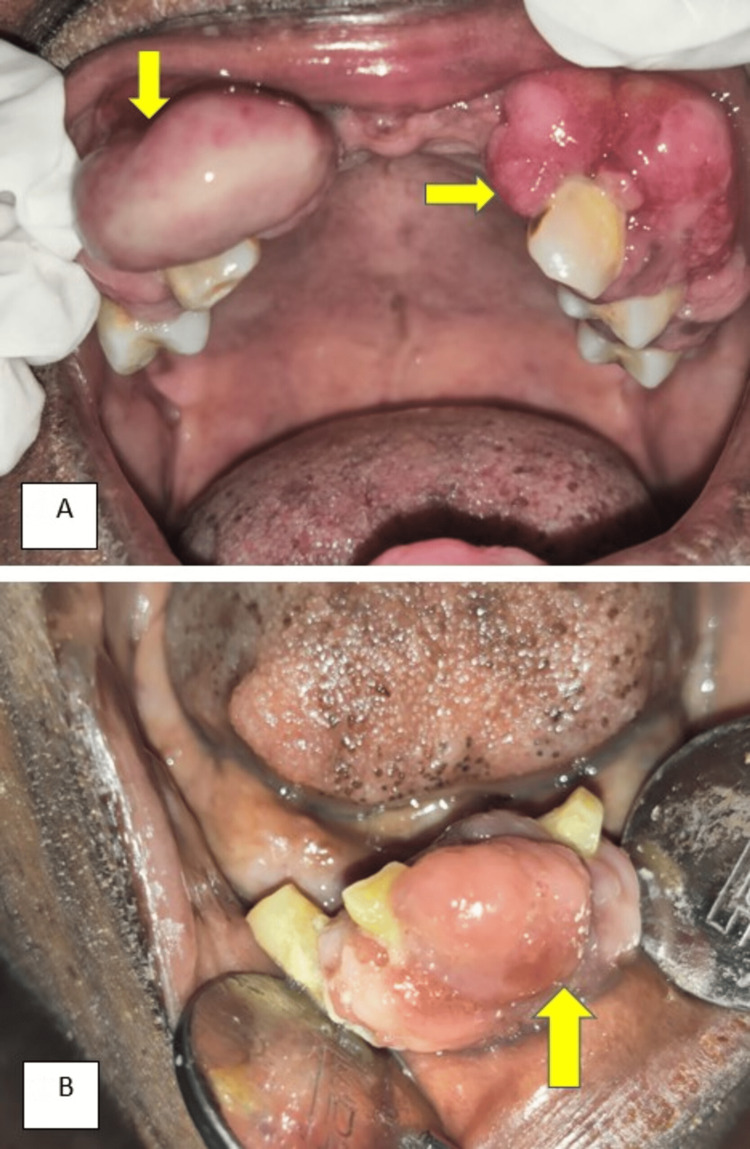
(A) Gingival overgrowth involving labial aspect of attached gingiva, interdental papilla in 13, 14, 23, 24, 25, 33, 42, and 43 tooth regions. (B) Gingival overgrowth involving labial aspect of 13, 14, 23, 24, 25, 33, 42, and 43 tooth regions.

A 54-year-old female presented for a routine dental checkup with asymptomatic gingival growth. Her past medical history revealed she is a known hypertensive and under antihypertensive medication amlodipine (5 mg) twice daily for four years. The gingival overgrowth started as a small one and had progressed to the present state. Her family did not have a history of similar symptoms. The oral hygiene status was poor and the Silness-Loe plaque index criteria revealed a score of three, due to the presence of large amounts of visible supragingival calculus around all the teeth present in 13, 14, 23, 24, 25, 33, 42, and 43 tooth regions. On intraoral examination, overgrowth of the gingiva is seen covering the labial aspect of the attached gingiva, and interdental papilla in 13, 14, 23, 24, 25, 33, 42, and 43 tooth regions (Figure 1).

**Figure 2:**
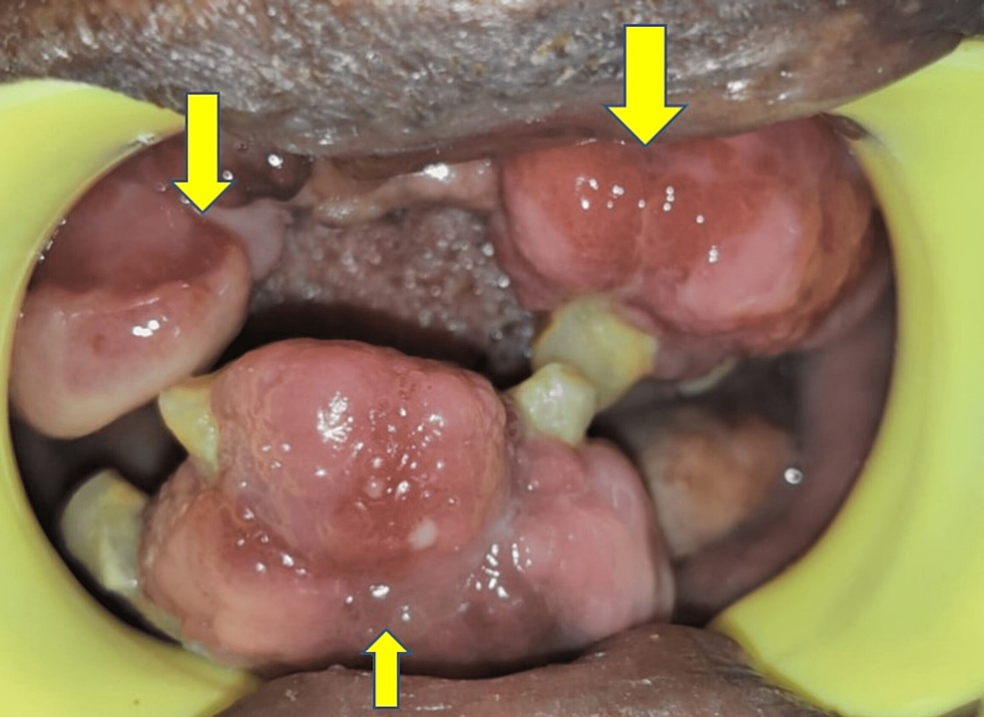
Gingival overgrowths interfered during occlusion.

On palpation, the gingival overgrowths were non-tender and firm in consistency. The gingival overgrowth interfered during occlusion (Figure 2). 

The differential diagnosis includes leukemic gingival enlargement. Clinically leukemic gingival enlargement bleeds spontaneously and spontaneous avulsion of the tooth by itself, which do not occur in drug-induced gingival enlargement. A haemogram analysis was done to rule out gingival enlargement caused secondary to leukemia or infections, the results of which are shown in Table 1.

**Table 1:** Hemogram report

Hematological parameters	Observed values	Normal ranges
Total WBC	9,400 cells/cmm	5,000-10,000/cmm
Neutrophils	64%	50-70%
Lymphocytes	29%	20-40%
Eosinophils	6%	1-6%
Monocytes	1%	1-4%
Erythrocyte sedimentation rate	13 mm/hr	0-20 mm/hr
Hemoglobin	10.8 gms %	12-16 gms%
RBC	3.89 millions/cmm	3.9 millions/cmm
Red cell distribution width	12.50%	11-16%
Platelets	3.43 lakhs/cmm	1.5-3.5 lakhs/cmm

The other treatment options for gingival overgrowth include cryotherapy, electrocautery, root planning, and flap surgery. Benidipine 2 mg twice daily was used in place of amlodipine because the patient declined treatment for the gingivectomy operation. The patient was also requested to undergo periodic follow-up dental visits. The patient was advised of strict oral hygiene instructions, such as the use of 0.2% chlorhexidine mouth rinses between her meals. Noncompliance and negligence in oral healthcare led to the patient not attending further dental treatment procedures.

## Discussion

Amlodipine is an oral dihydropyridine calcium channel blocker with structural similarities to nifedipine, which commonly causes gum hypertrophy [1]. Amlodipine has a long half-life of 30 to 50 hours [1]. Generally, amlodipine-induced gingival overgrowth is reported within three months of the drug's initiation when the dose is 10 mg/day [1]. In our patient, however, the enlargement occurred after four years of taking the drug at a dose of 5 mg twice daily. The time duration of gingival hypertrophy varies in our case due to multifactorial causes such as genetic susceptibility and host response to drug-induced gingival fibroblasts, interleukins, and matrix-metalloproteinases. Gingival hypertrophy caused by amlodipine is no longer rare [2]. Other significant risk factors include genetic susceptibility, drug dosage, and treatment duration [2].

It is important to explain this adverse effect to patients as amlodipine is the most commonly prescribed drug for the long-term maintenance of hypertension and angina [3]. To prevent gingival hypertrophy, patients need to be informed about meticulous plaque control [3]. The physician also needs to be aware of the associated drug variables to manage these cases effectively [3]. The most reliable method of treating amlodipine-induced gingival overgrowth is a combination of surgical and non-surgical periodontal treatment with drug substitution [3]. An increase in extracellular matrix proteases and increased fibrotic tissue deposition is correlated with amlodipine's effect on modulating fibrosis response in gingival fibroblasts [4]. John K et al. reported a case of amlodipine-induced gingival enlargement in a 19-year-old male with stage 5 chronic renal disease [5].

The substitution of amlodipine with benidipine is beneficial in suppressing amlodipine-induced gingival hypertrophy by decreasing lymphocytes expressing delayed rectifier potassium channels [6]. Ocumus OF suggested the use of a 445 nm blue diode laser in treating amlodipine-induced gingival enlargement, this laser also causes fewer thermal side effects than traditional high-wavelength diode lasers [7]. When gingival tissue grows, it can create pockets that are far from reach for a toothbrush or dental floss, impairing optimal oral hygiene and making the host more susceptible to oral infections, cavities, and periodontitis [8]. As the first step in managing drug-induced gingival hypertrophy, it is necessary to stop the offending drug and replace it with an alternative [8]. Maintaining good oral hygiene practices, including professional plaque cleaning [8]. To overcome adverse effects of complications such as stroke and angina, one must not stop the calcium channel blocker [8].

## Conclusions

Gingival hypertrophy caused by certain drugs, including amlodipine, may occur in genetically susceptible individuals. There is no clear explanation for the mechanism behind gingival hypertrophy, but a multifactorial theory has been proposed that unifies the phenomenon. In addition to causing difficulty with speech and mastication, gingival hypertrophy also contributes to poor oral hygiene and unaesthetic appearance. We describe the case of a 54-year-old female who developed gingival hypertrophy due to the long-standing antihypertensive medication amlodipine 5 mg taken for four years. Dentists, general physicians, and cardiologists must be well versed in the clinical presentation of amlodipine-induced gingival enlargement and must not stop the calcium channel blocker as it may lead to deteriorating life-threatening complications such as stroke secondary to uncontrolled systemic hypertension or cardiac angina. 
